# Learning mechanisms influencing infants’ early socio-pragmatic abilities

**DOI:** 10.1098/rstb.2023.0487

**Published:** 2025-08-14

**Authors:** Gideon Salter, Colin Bannard, Silke Fricke, Emily Hancock, Penny Levickis, Antonia Pavlou-Rodriguez, Julian Pine, Kiera Solaiman, Emma Smith, Emma Thornton, Molly Willis, Danielle Matthews

**Affiliations:** ^1^ School of Psychology, The University of Sheffield, Sheffield S1 4DP, UK; ^2^ Department of Psychology, University of York, York YO10 5DD, UK; ^3^ Department of Linguistics and English Language, The University of Manchester, Manchester M13 9PL, UK; ^4^ Division of Human Communication Sciences, School of Allied Health Professions, Nursing and Midwifery, The University of Sheffield, Sheffield S10 2TS, UK; ^5^ Centre for Research in Effective Education in Early Childhood, Melbourne Graduate School of Education, University of Melbourne, Melbourne, Victoria 3010, Australia; ^6^ Department of Psychology, University of Liverpool, Liverpool L3 5TR, UK; ^7^ Manchester Institute of Education, The University of Manchester, Manchester M13 9PL, UK

**Keywords:** infancy, communication, pragmatics, responsiveness, parent–child interaction, socio-economic circumstances

## Abstract

Advanced pragmatic skills are hypothesized to depend on early experience of interaction. However, we do not yet fully understand the causal pathways involved. In the current study, we explored one potential early learning mechanism by assessing whether increasing caregiver responsiveness to infant communication in turn promotes infants’ pre-linguistic communicative acts. In the first wave of a larger randomized controlled trial study, when their infants were around six months old, carers were randomly assigned to either a communication intervention or an active control intervention focused on physical health. When infants turned 12 months, home videos (*N* = 125, 64 active control intervention, 61 communication intervention) were analysed for infant pre-linguistic acts, and caregiver responses to infant pre-linguistic communication. We also examined whether these variables varied by socio-economic circumstances. Pre-registered analyses indicated that the intervention led to increases in infant communicative acts and caregiver semantically contingent responses to infant communicative behaviours. This indicates that the experience of communicating with a responsive caregiver has a causal effect on the development of the infant’s pre-linguistic pragmatic skills that are thought to provide the basis for later language, pragmatics and Theory of Mind.

This article is part of the theme issue ‘At the heart of human communication: new views on the complex relationship between pragmatics and Theory of Mind’.

## 1. Introduction

The roots of both pragmatic and Theory of Mind (ToM) abilities are thought to be found in social interactions during human infants’ first year of life. These involve infants and carers responding to each other and jointly attending to features of the world around them [1–3]. Pragmatic abilities involving conventional language build on the earlier abilities to (i) use vocalizations and gestures to direct an interaction partner’s attention and (ii) understand communicative intentions [2–4]. Likewise, ToM abilities, involving understanding others’ conflicting perspectives on the world, build on the earlier sensitivity to what has and what has not been in joint attention [5,6]. To understand the relationship between these domains, then, it can help to gain a clearer view of their origins. In particular, understanding the early experiences and learning mechanisms that shape infant pre-linguistic communicative abilities is crucial for understanding the developmental trajectory that, via the emergence of language, culminates in both advanced pragmatic and ToM abilities.

Variation in early social experience can come from a range of sources, some of which are nested within each other [7]. One source is variation in caregiver responsiveness. The consistency and manner in which carers respond to their child is widely understood to be a major influence on child language development [4,8–10]. However, it is unclear whether caregiver responsiveness influences infants’ intentional *pre-linguistic* communication to the same extent. A broader source of variation in early social experience stems from the socio-economic circumstances (SEC) that infants are born into and experience as they grow up. Language outcomes are associated with SEC, and this is likely to be, at least partly, explained by caregiver responsiveness [11]. However, the extent to which the SEC influences pre-linguistic interactions is also not clearly established. Investigating how differences in early social experience influence the early stages of infant communicative development is important for understanding the extent to which infants might already be drawing upon information in their social environment and using this to guide their communication, well before complex forms of social reasoning or advanced pragmatic abilities have emerged. Addressing these issues will contribute to a more clearly specified picture of whether and how participation in early social interactions provides the first steps on the path to complex pragmatic and ToM abilities.

This article draws upon data from a larger randomized controlled trial (RCT) with a focus on promoting early language development by supporting caregiver responsiveness (see [12,13]). The aim of this article is to assess whether intervening to increase caregiver responsiveness has a causal impact on infant pre-linguistic communication. It will also explore whether these effects vary by SEC.

### (a) Interaction and the socio-pragmatic origins of language

A set of early proto-pragmatic communicative behaviours have been argued to signal the readiness of human infants to make the transition to using conventional language and other areas of advanced social cognition, including the perspective-taking involved in ToM [6]. Referential gestures (including showing, giving and index-finger pointing) are widely understood to be of particular importance as behaviours that are used to share attention to features of the world in a manner that is later supplemented by verbal reference (e.g. [3,4,14–17]). As well as reflecting infants’ emerging socio-pragmatic capacities, they may also be effective at eliciting caregiver responses, which may in part explain their associations with later language. Vocal markers include the age of onset of infant babbling [18,19] and the rate of canonical babbling expressed as a proportion of all syllables at 10 months [20]. Their relation to later language is at least in part due to the emerging articulatory requirements of babbling and speech, but may also be due to the role of babbling in eliciting caregiver responses [19]. At 12 months, a particularly strong vocal predictor of later language is infants’ production of gaze-coordinated vocalizations (producing a vocalization and looking at the carer’s face within 1 s). This has been taken to suggest that infants’ intentionally communicative use of vocalizations serves as a key developmental step towards language, perhaps because they indicate motor and social-cognitive readiness on the part of the infant and/or because these behaviours are particularly effective in eliciting rich caregiver responses [4].

Caregiver responsiveness is widely believed to be a key mechanism influencing the trajectory of infant socio-pragmatic development. Numerous studies have highlighted the importance of caregiver responsiveness for infant social development, with a particular focus being language development (e.g. [4,10,21–23]). Three types of responses have received attention. First is carers’ *semantically contingent talk*—talk that is contingent on the child’s activity and relevant to their attentional focus [3,10,11,24]. This talk can be produced as a ‘running commentary’ on infants’ activity or in response to infant communicative behaviours, with the latter type having been found to be especially predictive of language development [4,9]. Second, is carers’ *vocally imitative responses*. Carers’ vocal imitation of their 13-month-old infants has been found to predict later language outcomes [25], possibly because it reinforces an infant’s sense of communicative efficacy while also (in the case of imitated words) ensuring that the conversation is semantically related to their activity. Third, carers’ vocal responses may be promptly produced in response to an infant’s communicative behaviour but neither semantically contingent nor imitative (henceforth referred to as ‘*other vocal responses*’). Simply responding vocally in any way to an infant’s act is arguably beneficial to infants’ communicative development. The work of Goldstein and colleagues [8,26,27] has demonstrated that nine-month-old infants adjust their vocalizations as they receive vocal feedback from their carer, while five-month-old infants expect their babbling to be met with a contingent vocal response by familiar and unfamiliar adults. These studies suggest that when infants vocalize, receiving even a generic vocal response from a caregiver can encourage further infant vocalization and encourage the infant to expect a response. It is plausible that a similar process occurs for responses to communicative gestures.

The current study, building on the above observational evidence, aimed to test whether three types of caregiver response can be promoted through early carer-based intervention, and whether these changes have a causal effect on infant communication.

### (b) Socio-economic circumstances and early interaction

Individual differences in infants’ experience of early interaction are thought to explain, at least in part, why their early language skills tend to vary as a function of SEC [21,28]. A social gradient in language ability has been observed to emerge in infants’ second year in both the US and the UK [11,29], and studies have sought to examine the source of these differences in early social interactions. Existing work in this area suggests that carers’ semantically contingent talk, but not infant pre-linguistic communicative behaviours, varies according to SEC. For example, McGillion and colleagues [11] found that there were already differences in carers’ production of semantically contingent talk when the infants in their study were 11 months old, but that there were no such differences in the infant communicative behaviours they measured (vocalizing and pointing). However, there are a number of issues relating to SEC-based differences in infant–caregiver interaction that require further exploration.

First, to date, when it comes to gestural communication, only variation in the production of index-finger pointing has been examined, and it has been found that there are no SEC-based differences in index-finger pointing at 11 months [11]. However, pointing occurs with relatively low frequency at this age and it may be that SEC-based differences arise in earlier emerging communicative behaviours, such as showing gestures and giving gestures. These behaviours, alongside gaze-coordinated vocalizations, have been shown to be strong predictors of later vocabulary outcomes [4], but are underexplored with respect to SEC.

Second, while we know that carers’ semantically contingent talk in general relates to SEC during infancy [11], recent evidence suggests that it is specifically semantically contingent talk produced in response to an infant’s communicative act that is most valuable as a predictor of later language [4]. This is likely to be in part due to the fact that the latter measure is a dyadic measure that necessarily also captures infant ability and motivation. However, it is arguably also predictive because it reflects the value of infants’ receiving well attuned input at a moment when they are motivated to communicate about the very thing that input relates to. We do not yet know whether production of such behavioural sequences varies according to SEC, nor is it clear whether imitative or other vocal responses vary in this way.

Addressing the above questions is important not only for theory building but also for designing early interventions to support the development of language skills, be it to address the risk of delay associated with socio-economic disadvantage [11,30] or to support children identified with low language skills (e.g. [31,32]). These interventions have focused on different stages of development, from infancy to the early school years. Relatively few interventions, particularly among those targeted at mitigating the risk of language delay associated with social disadvantage, have started during infants’ first year, with fewer still measuring infant pre-linguistic outcomes or directly testing the role of infant–caregiver interaction (though see [33–35]. Other studies with slightly older infants suggest it may be possible to promote the frequency of infant gestural communication, specifically index-finger pointing, although results are mixed on this front [36,37].

While the above interventions are promising, none of them permits us to test the causal hypotheses regarding caregiver responsiveness put forward above. It is possible that promoting caregiver responsiveness from very early on not only would benefit word learning but also could have a causal effect on infants’ pre-linguistic communication by 12 months. In principle, this would then feed into a positive cycle of interaction, with infants communicating more often and, in turn, carers responding more often.

### (c) Summary and the current study

Advanced linguistic, pragmatic and ToM abilities are built on a foundation of pre-linguistic social interactions that typically take place during infants’ first year. However, the relation between pre-linguistic communicative acts and caregiver interaction and responsiveness at this early stage is still not well understood. To the best of our knowledge, no previous work has explored causal effects on gestures other than pointing, or carers’ responses to gaze-coordinated vocalizations and gestures, even though some such specific behaviours have recently been identified as highly predictive of later language development. While interventions have proved effective in promoting language outcomes and caregiver responsiveness from 14 months [11,34,38–40], the extent to which early interventions promote earlier interaction and infants’ pre-linguistic communicative acts remains unclear.

To address these questions, we analysed the data from a recent RCT [12] in more depth in order to test whether early experience of interaction with a responsive caregiver drives pre-linguistic pragmatic development. The RCT involved delivering an early communication intervention (or active control intervention based on dental health, diet and motor development) to families from varied socio-economic backgrounds across the four nations of the United Kingdom (England, Wales, Scotland and Northern Ireland). Carers were texted short videos from the Tiny Happy People service (or matched control videos) three times a month from the point when they started the intervention (median: six months) until outcomes were measured at 12 months. The impact of the intervention on interaction and pre-linguistic communication was assessed by collecting home video recordings made by carers on their phones, allowing direct assessment of interactions in a naturalistic context [41,42].

Home video recordings were coded in two ways. First, we coded 12-month-old infants’ production of pre-linguistic communicative acts that are known to be positive predictors of later language (showing, giving and index-finger pointing gestures and gaze-coordinated vocalizations). Second, we coded how often these pre-linguistic communicative acts and vocalizations without gaze coordination were responded to by carers with response types that are known or hypothesized to be positive predictors of later language (semantically contingent responses, imitative vocal responses or other vocal responses). We predicted that the communication intervention would have a positive effect on caregiver responsiveness and infant pre-linguistic communication, in line with previous work demonstrating the effectiveness of remotely delivered caregiver interventions [43]. We also expected that, if anything, these effects would be greater for families experiencing socio-economic disadvantage, in line with previous work showing that socially disadvantaged families benefit more from an intervention targeting caregiver responsiveness [11].

## 2. Methods

### (a) Design

In this RCT, with a parallel group design, families were assigned to one of two arms: arm 1: allocated a communication intervention; or arm 2: allocated a closely matched active control intervention that promoted physical development (healthy eating, dental health and motor development). The RCT was pre-registered on the Open Science Framework (OSF) (https://osf.io/bzv57) and ClinicalTrials.gov (NCT04919343), and information regarding the randomization process can be found in the OSF pre-registration. See electronic supplementary material, file S1 for CONSORT diagram. For the paper focusing on the full RCT, see Matthews *et al.* [12,13]. The present article draws upon a subset of participants who took part in the full RCT (for details, see below) and who submitted an optional home video recording of an interaction between them and their infant. The analyses of caregiver and infant behaviours at 12 months that were conducted on the data collected from this subset were pre-registered separately on OSF [44].

### (b) Participants

Participants were the subset of those participating in the full RCT who chose to send in home videos when infants were 12 months old. In the full RCT, 435 families were recruited and randomized. One family withdrew from the study after the randomization process, leaving 434 families. Carers were invited to send video recordings of free play featuring them and their 12-month-old infant, an optional aspect of the study that was not remunerated. In total, 150 families sent in a video (see below for further details). Inclusion criteria for participation in the RCT were (i) babies were aged four to nine months at the time of recruitment; (ii) born at full term (not three weeks or more premature); (iii) birth weight of ≥2.5 kg (5 lb 8 oz); (iv) the main language used at home was English (≥80% of the time); (v) postcode was within the lowest 5 deciles (1–5) of the Office of National Statistics Index of Multiple Deprivation (IMD [45]); (vi) carers had access to the Internet and a mobile device that would enable them to receive text links and watch videos; and (vii) neither the caregiver nor the infant had a condition known to affect child language development.

Note that the IMD is a composite measure that collapses across a number of neighbourhood dimensions (e.g. levels of education, employment, crime) to rank postcodes from most to least disadvantaged. Indices are specific to each UK country (England, Wales, Scotland and Northern Ireland) but are composed in a similar way. Postcodes are specific to the road a person lives on and so are a useful proxy for socio-economic circumstances. We used IMD to selectively recruit families who were not in the top 50% most advantaged areas of the country based on postcode so as to avoid having a sample skewed to more advantaged families (which tends to happen by default when recruiting to research studies). Having recruited families, we then collected baseline demographic information including a more accurate measure of one dimension of family SEC, namely primary caregiver education. This measure is known to be the best SEC predictor for child language ability in the UK [46], which is why we used it for the analyses.

For the 150 families who provided video recordings, inclusion criteria were applied to gauge ‘codability’ depending on whether a given outcome measure relied on the visibility of the caregiver or the infant. For full details regarding participant inclusion, as well as condition allocation and demographic information, see electronic supplementary material, file S2. For the coding of individual infant communicative acts and caregiver responses to them, 125 families (61 intervention, 64 control) were included in the final sample.

Since the current study relied on carers from the wider RCT opting to send in videos, we could not directly determine the sample size. However, as a guide, we nonetheless conducted a power analysis based on the expected rate of production of infant pre-linguistic communicative acts (an estimated four pre-linguistic communicative acts per 5 min video recording in the active control condition, based on counts in [4]). To detect an increase in pre-linguistic communicative acts with a small effect size (0.2), corresponding to an increase of 1.43 additional behaviours, and employing the pre-registered analysis plan, a sample of 57 participants would be required to detect an effect with 80% power.

### (c) Procedure

The entire trial was conducted remotely and commenced during the COVID-19 pandemic, with no face-to-face contact. For both study arms, the intervention started when the infants were around six months (median: six months; range: four to ten months). The format for each intervention was identical except for the video content. In the communication intervention condition, three times a month (roughly every 10 days), carers were sent SMS texts with a link to publicly available age-appropriate video resources produced as part of the ‘BBC Tiny Happy People’ service (and on average, they clicked on just over a third of these; see electronic supplementary material, file S3). These video resources were produced with the aim of supporting language development, and covered topics including the three types of responsiveness analysed here, namely responding vocally to babies’ babble, imitating infant communication and providing semantically contingent talk. Carers were encouraged to integrate activities from the resources into their everyday routine. In the active control intervention condition, the same schedule of texts was followed, but the videos were about topics such as healthy eating, physical activity and dental care. For further specific examples of the texts and video resources that were sent to carers, see electronic supplementary material, file S3.

When the infants were approaching 12 months of age, carers were sent a follow-up questionnaire. Responses to this questionnaire were not analysed for the present article, but for transparency we note that these measured caregiver self-efficacy, caregiver self-affirmation, and the acceptability of the intervention materials. Carers were also invited to send a 5 min video involving them and their infant playing as they would typically. They were asked to ensure that both they and their infant were visible on the recording (made, in most cases, with their mobile phone). No further instructions (e.g. regarding the type or quantity of objects used in play) were provided. Carers made the recordings in their own time, rather than in a call with a researcher [47], and video recordings were sent via secure online transfer (WeTransfer or Google Drive). A recent (2022) survey of adults aged 18 to 64 years (*n* = 4034) found that 97% of households in the UK have access to a smartphone [48], suggesting that this is a viable avenue for data collection even for low-income households in the UK.

### (d) Coding

To measure individual acts of infant and caregiver communication in depth, four coders who were blind to participants’ condition allocation coded the videos. All 125 videos were coded by two different coders following the guidelines in electronic supplementary material, file S4. Two categories of behaviour were coded, one at the level of the infant and one at the level of the infant–caregiver dyad (based on [4]). At the infant level were pre-linguistic communicative acts—communicative behaviours produced by infants that have previously been identified as positive predictors of later language skills. The behaviours falling under the category of pre-linguistic communicative acts are listed in table 1 and defined in file S4. The coders identified each instance of these four behaviours, which were then summed to provide the variable *total frequency of pre-linguistic communicative acts*. A total frequency count was preferred owing to the inconsistency with which infants produce any single one of these behaviour types during free play (like words, occurrences of these behaviours tend to clump together, a phenomenon known as ‘burstiness’ [49]; see also [4,50]), combined with the relatively short length of the video recordings.

**Table 1.** Median, range and interquartile range for all measures of infant communication and caregiver responsiveness.

outcome variable	median	minimum	maximum	IQR
pre-linguistic communicative acts	2	0	12	4
semantically contingent responses^a^	4	0	24	5
imitative responses^a^	1	0	9	2
other vocal responses^a^	2	0	14	4

^a^ Based on Donnellan *et al*. [4], response measures include responses to both pre-linguistic communicative acts (give, show, pointing gestures and gaze-coordinated vocalizations) and, additionally, vocalizations that are not gaze coordinated. Therefore, the number of responses may exceed the number of pre-linguistic communicative acts for some participants.

At the dyad level were a set of measures that relied on the infant producing a communicative act and the caregiver producing some kind of vocal response within 1 s. The three response types are listed in table 1 and defined in electronic supplementary material, file S4. Based on what Donnellan *et al*. [4] found to be predictive of later language, the coders recorded caregiver vocal responses (i) to all pre-linguistic communicative acts noted above, and additionally (ii) to vocalizations produced by infants that were not gaze-coordinated (since Donnellan *et al.* found that the latter combination was also a positive predictor of vocabulary outcomes even if the vocalizations alone were not).

All four of the behaviour categories that were used as outcome variables (the total count of infants’ pre-linguistic communicative acts, and the total counts for each of the three dyadic measures) were assessed for reliability using the generalized concordance correlation coefficient [51]. Agreement ranged from good to excellent (Intraclass Correlation Coefficient (ICC) range = 0.71–0.88). For further details, see electronic supplementary material, file S5.

## 3. Results

### (a) Participant demographic information

Electronic supplementary material, file S2 provides demographic details for the children in this study and for the full RCT sample. The participants who provided the video data analysed here did not differ substantially from the main RCT sample (*n* = 435), except that participants who sent in videos were slightly more likely to have a degree. To explore this further, analyses taking into account caregiver education are included below. For 123 participants (98%), the mother was the primary carer, with one father participating, and one participant not recording this information. The sample was ethnically representative for the United Kingdom.

### (b) Descriptive statistics

Table 1 displays the descriptive statistics for each outcome variable. For descriptive statistics split by (i) condition and (ii) caregiver education, see electronic supplementary material, fileS6.

### (c) Effect of the intervention on infants’ pre-linguistic communicative acts

**Figure 1.**
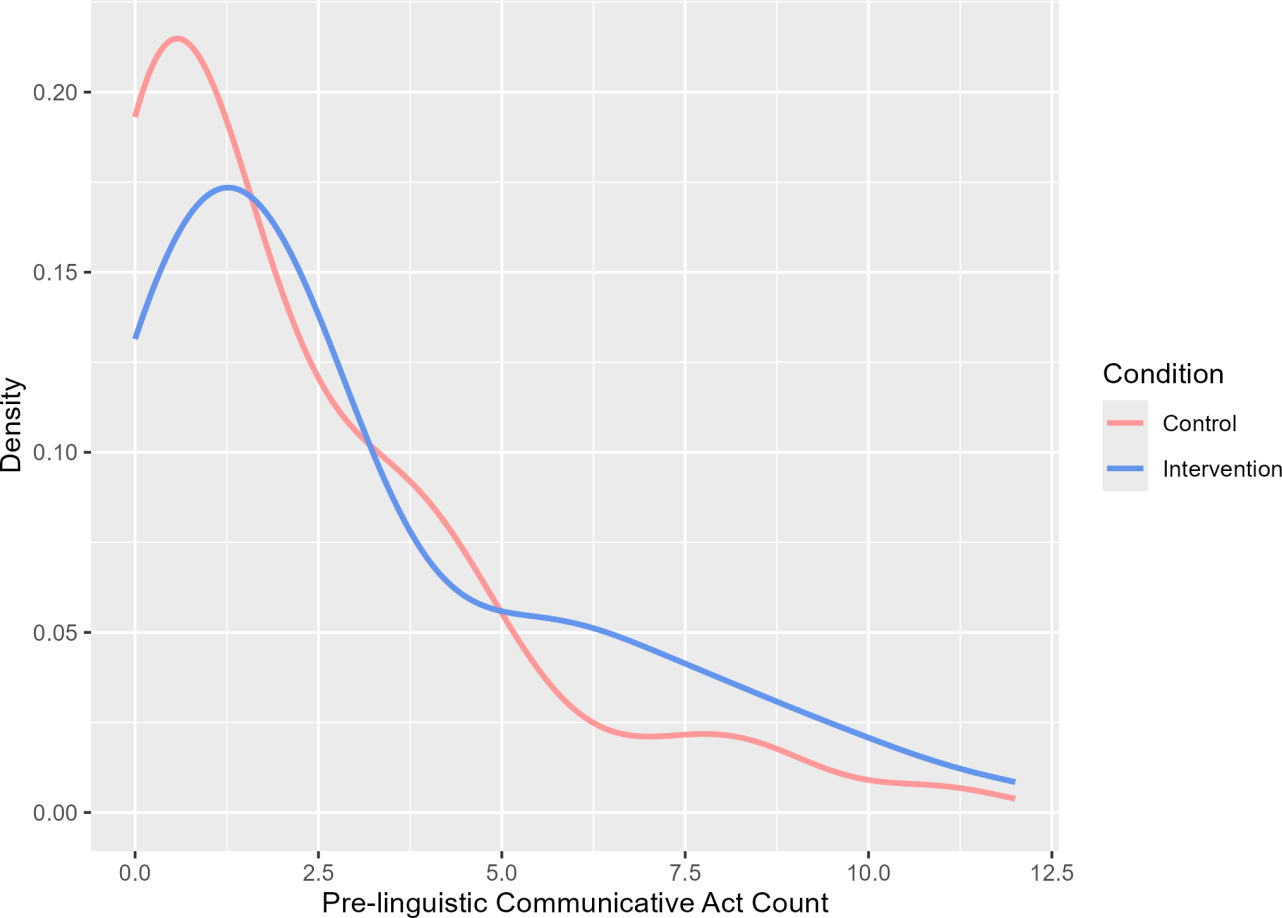
Density curves for infants’ pre-linguistic communicative acts as a function of condition.

The first set of analyses focuses on infants’ production of pre-linguistic communication acts. Figure 1 displays the probability density for the frequencies of production of pre-linguistic communicative acts in each condition. These indicate the best estimate given the data of the probability with which a participant or dyad has a given count for a given behaviour or response in each condition. The curves for pre-linguistic communicative acts for the two conditions diverge, such that the probability of lower counts is greater in the active control condition.

For the models assessing infants’ production of pre-linguistic communicative acts and carers’ responses, the outcome variables were counts, and, following pre-registered tests for over-dispersion [52], negative binomial regressions were used to model the data. To aid in the interpretation of interactions, both predictor variables (SEC and condition) were effect-coded as −0.5 and 0.5. For the full table of model coefficients, see electronic supplementary material, file S7. As we had pre-registered directional predictions for each analysis, we followed recent guidelines that encourage the use of one-tailed tests in such cases [53,54].

In order to test the hypothesis that infants whose families took part in the communication intervention would produce more pre-linguistic communicative acts at 12 months than children whose families took part in the active control intervention, a pre-registered negative binomial regression model with the count of pre-linguistic communicative acts as the outcome variable and condition as the predictor variable was fitted to the data. To account for differences in the final length of recording (ranging between the lower limit of 240 s and the upper limit of 300 s), the length of time the infant’s behaviour was codable during the recording was an offset variable in the model. The model did not identify a significant effect of condition (*β* = 0.32, *p* = 0.054, one-tailed). An exploratory analysis that added caregiver education into this model showed a similar pattern of results.

In order to test the hypothesis that the effect of intervention on infants’ production of pre-linguistic communicative acts would be moderated by caregiver education, a pre-registered negative binomial regression model with the count of pre-linguistic communicative acts as the outcome variable, condition, caregiver education and their interaction as predictor variables, and codable time as an offset variable was fitted to the data. The term for condition was significantly above zero (*β* = 0.449, *p* = 0.023, one-tailed). The interaction between condition and caregiver education was not statistically significant (*p* = 0.089, one-tailed), but the slope had a negative sign (*β* = −0.60). Taken together, these can be tentatively taken to indicate that the intervention may have an effect in households where the primary caregiver does not have a degree, with this effect being smaller or absent in households where the primary caregiver has a degree. There was no observed effect of caregiver education on pre-linguistic communicative acts (*β* = 0.11, *p* = 0.30, one-tailed).

### (d) Effect of the intervention on carers’ vocal responses

The second set of pre-registered analyses investigated differences in the three types of caregiver response.

#### (i) Effect of the intervention on carers’ semantically contingent responses

**Figure 2.**
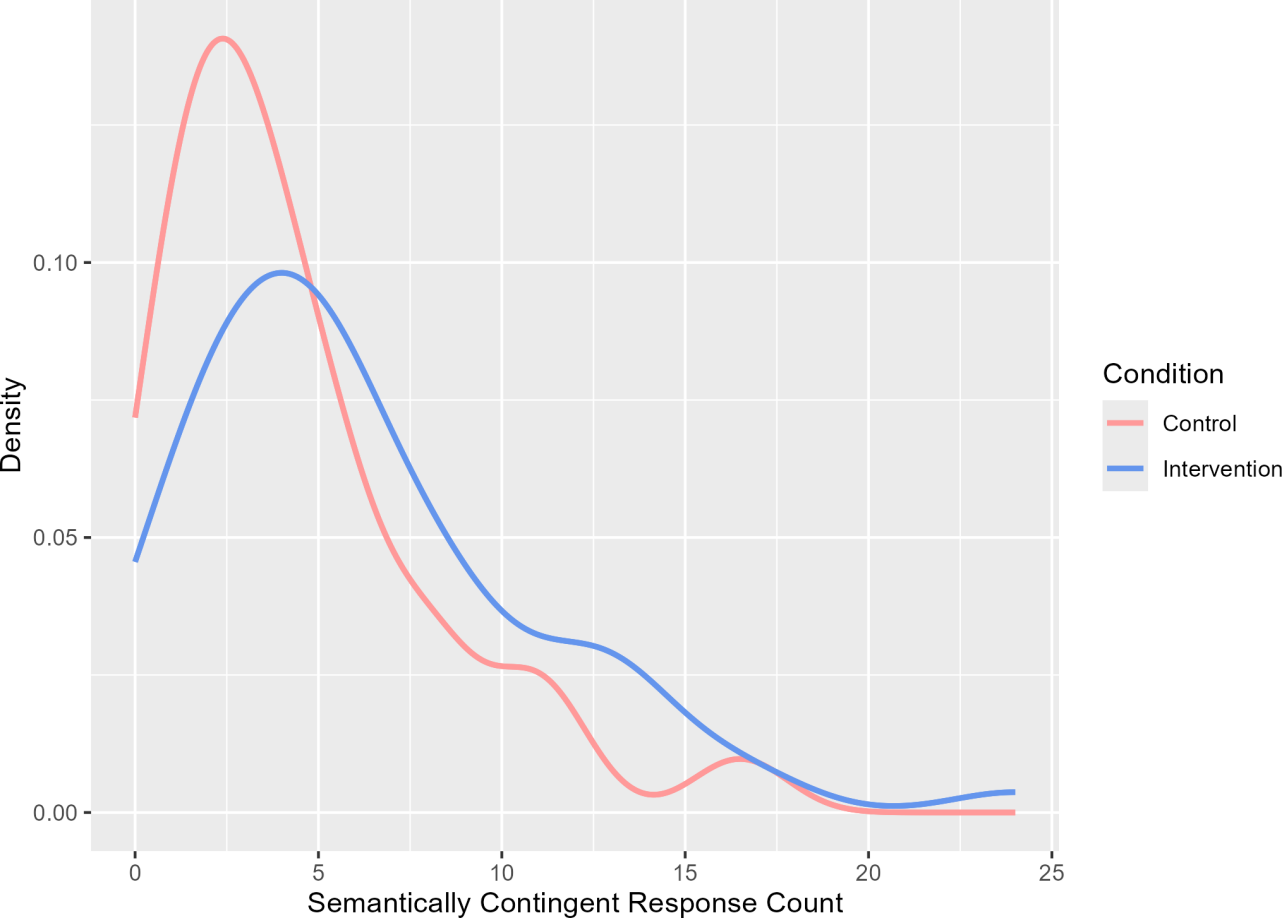
Density curves for carers’ semantically contingent responses as a function of condition.

Figure 2 displays the probability density curves for the frequency of production of semantically contingent responses. Semantically contingent responses for the two conditions diverge, such that the probability of lower counts is greater in the active control condition.

In order to investigate whether the intervention increased the number of times carers responded to their infants’ communicative bids in a semantically contingent way, a pre-registered negative binomial regression model with the count of caregiver semantically contingent responses as the outcome variable, condition as the predictor variable and codable time as an offset variable was fitted to the data. The model revealed a statistically significant effect of condition (*β* = 0.34, *p* = 0.009, one-tailed), such that carers produced more semantically contingent responses in the communication intervention condition than in the active control condition. According to model estimates, a caregiver in the communication intervention condition would produce 1.79 additional semantically contingent responses in 5 min compared with a caregiver in the active control condition (i.e. a 40% increase over a rate of 4.48 per 5 min in this dyad-based measure). An exploratory analysis that added caregiver education into this model showed a similar pattern of results.

In order to investigate whether differences in the counts of carers’ semantically contingent responses in the intervention group would be greater in carers without a degree, a pre-registered negative binomial regression with the count of caregiver semantically contingent responses as the outcome variable, condition, caregiver education and the interaction between them as the predictor variable, and codable time as an offset variable was fitted to the data. The model revealed a statistically significant effect of condition (*β* = 0.44, *p* = 0.003, one-tailed), such that carers produced more semantically contingent responses in the communication intervention condition. Caregiver education was also a statistically significant predictor (*β* = 0.34, *p* = 0.018, one-tailed), such that, according to model estimates, a caregiver with a degree would produce 1.64 additional semantically contingent responses during a 5 min recording (i.e. a 40% increase). Finally, the interaction between condition and caregiver education was significant (*β* = −0.53, *p* = 0.050, one-tailed). The negative estimate for the interaction term indicated that the effect of the communication intervention was smaller for participants with a degree.

#### (ii) Effect of the intervention on carers’ imitative vocal responses

**Figure 3.**
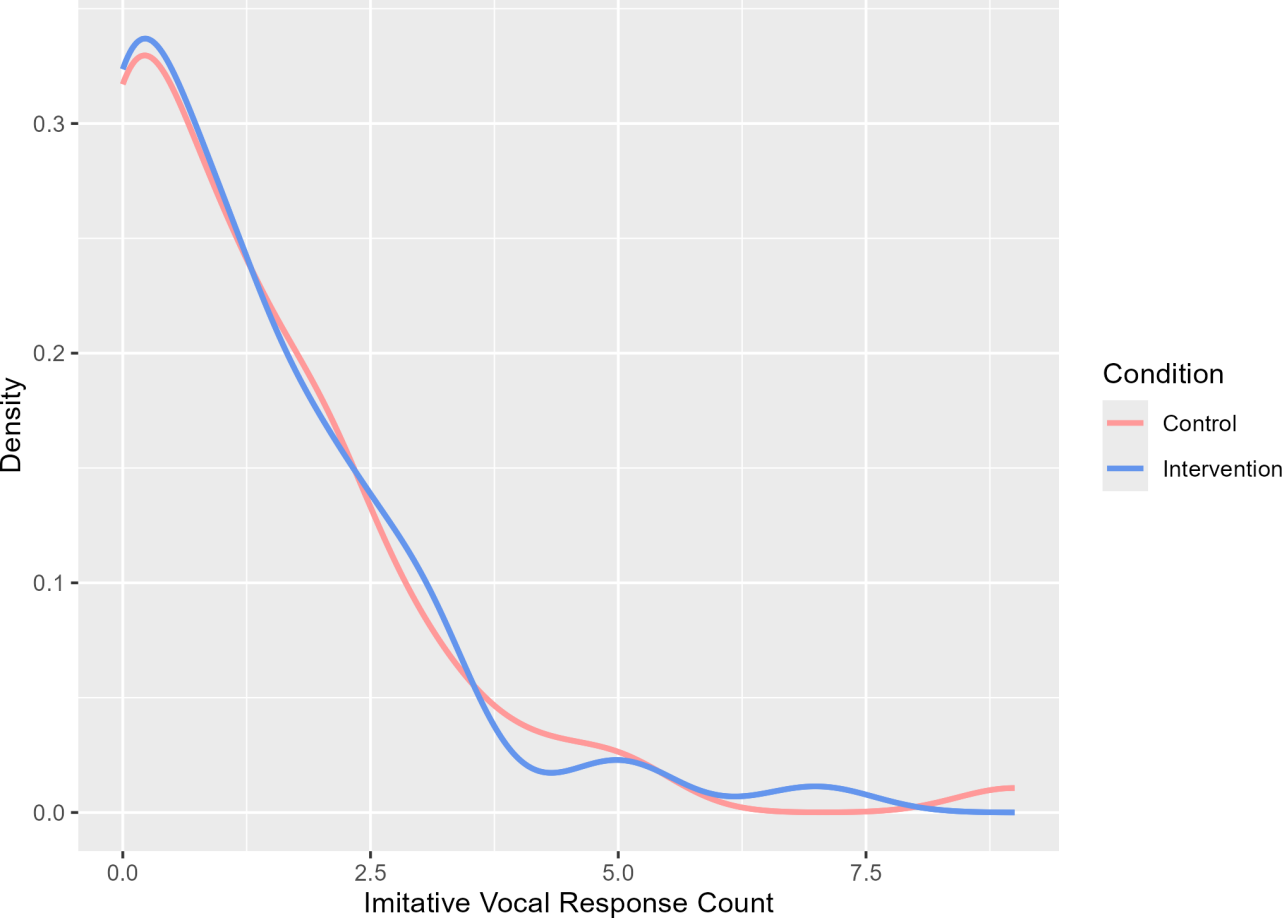
Density curves for carers’ imitative vocal responses as a function of condition.

Figure 3 displays the probability density curves for the frequencies of production of imitative vocal responses. There is no divergence between the conditions for imitative vocal responses.

In order to investigate whether the intervention increased the number of times carers responded to their infants’ communicative vocalizations with a vocally imitative response, a pre-registered negative binomial regression with the count of caregiver imitative vocal responses as the outcome variable, condition as the predictor variable, and codable time as an offset variable was fitted to the data. The model revealed no significant effect of condition (*β* = −0.05, *p* = 0.411, one-tailed) for imitative vocal responses. Likewise, there was no effect of caregiver education (*β* = 0.04, *p* = 0.431, one-tailed) and no interaction (*β* = −0.64, *p* = 0.090, one-tailed).

#### (iii) Effect of the intervention on carers’ other vocal responses

**Figure 4.**
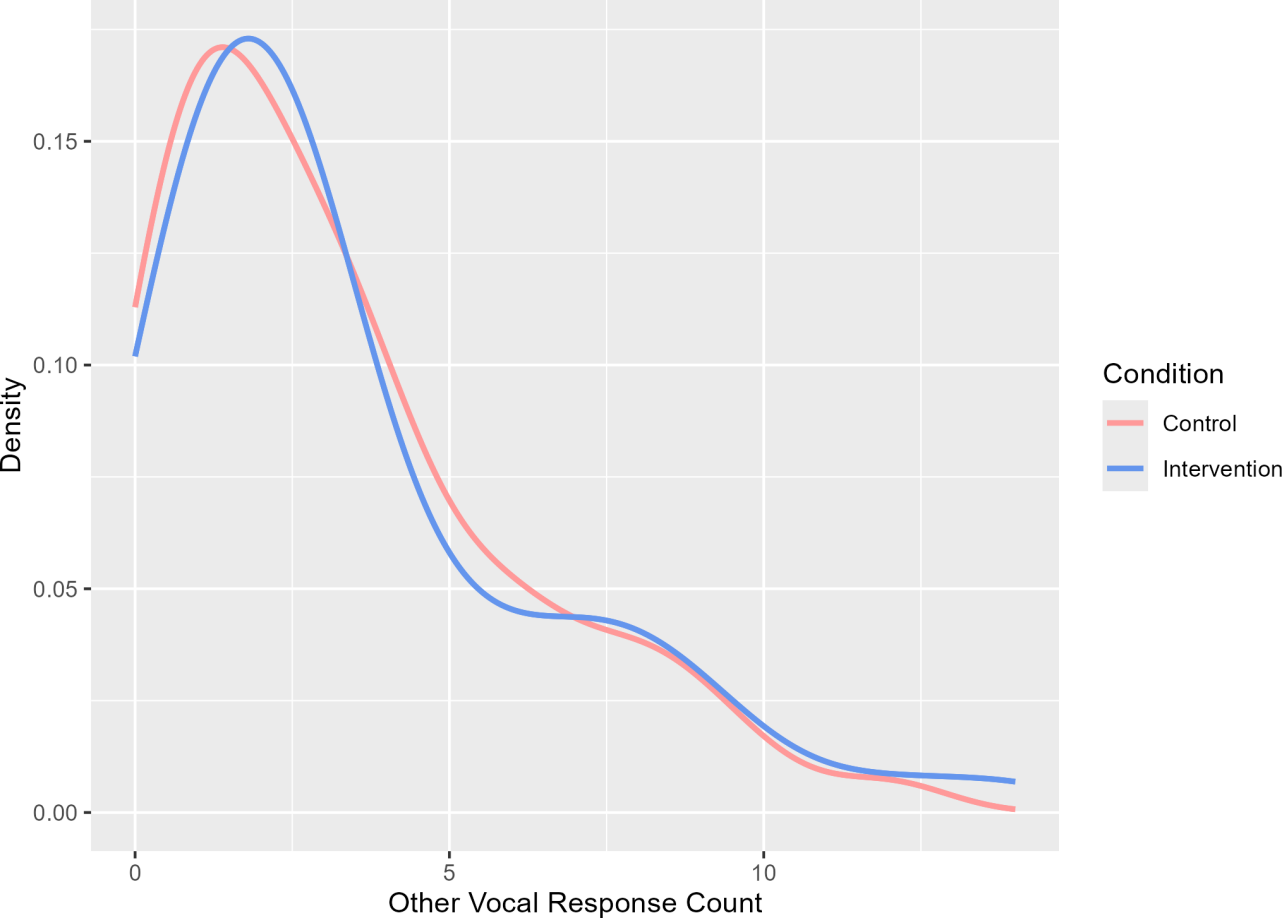
Density curves for carers’ other vocal responses as a function of condition.

Figure 4 displays the probability density curves for the frequencies of production of other vocal responses. There is no divergence between the conditions for other vocal responses.

In order to investigate whether the intervention increased the number of times carers responded to their infants’ communicative bids with a vocal (non-imitative) response, a pre-registered negative binomial regression with the count of carers’ other vocal responses as the outcome variable, condition as the predictor variable, and codable time as an offset variable was fitted to the data. The model revealed no statistically significant effect of condition (*β* = 0.06, *p* = 0.360, one-tailed) for other vocal responses.

In order to investigate whether differences in the counts of carers’ other vocal responses in the intervention group would be greater in carers without a degree, a pre-registered negative binomial regression with the count of carers’ other vocal responses as the outcome variable, condition, caregiver education and the interaction between them as the predictor variable, and codable time as an offset variable was fitted to the data. The model found no effect of condition on carers’ other vocal responses (*β* = 0.17, *p* = 0.164, one-tailed) or levels of education (*β* = 0.22, *p* = 0.102, one-tailed). However, the interaction between condition and caregiver education was marginally statistically significant (*β* = −0.57, *p* = 0.053, one-tailed). The negative estimate for the interaction term indicated that the effect of the communication intervention was smaller for participants with a degree.

## 4. Discussion

This study examined the causal effect of promoting early caregiver responsiveness on pre-linguistic pragmatic development. Delivering a phone-based service to carers (starting when their infants were around six months) resulted in an increase in infants’ production of pre-linguistic communicative acts and an increase in carers’ semantically contingent responses, but no changes in carers’ imitative and other vocal responses. Since there was a slight imbalance in the number of carers with a degree in each condition, we ran exploratory analyses (in addition to those pre-registered) to investigate whether intervention effects were similar when caregiver education was controlled for, and found effect sizes were similar in these analyses. As with previous studies, carers with a degree produced more semantically contingent responses than carers without a degree. There was a significant interaction between condition and caregiver education such that the effect of intervention was slightly greater for carers without a degree [11,21,22,55]. However, given the imbalance in the number of carers with versus without a degree, this effect would need to be replicated in a larger sample for us to be confident that it generalizes.

These findings suggest that promoting caregiver interaction and responsiveness had a causal effect on infants’ pre-linguistic communication. While the observed increases in caregiver responsiveness were plausibly driven by carers implementing the advice provided in the communication intervention, the mechanism through which the infants’ behaviour changed warrants further discussion. It is plausible that there is a dynamic relation between caregiver responsiveness and infant pre-linguistic communication; increasing how often carers respond encourages infants to produce more communicative behaviours. While the logic of a positive feedback loop could apply to any response to infant communication, it appears that only semantically contingent responses were promoted and are thus assumed to drive the observed differences in infant communication. This suggests that there is something particularly beneficial or rewarding about receiving a semantically contingent response to a communicative attempt—and this promotes subsequent infant pre-linguistic communication above and beyond receiving other kinds of vocal response.

There are several options as to what this ‘something’ could be. First, semantically contingent responses are typically longer (they include more words) than other vocal responses. An explanation that focuses solely on the sheer quantity of linguistic input is unlikely to provide the whole story, given previous evidence that caregiver linguistic input quality better explains variation in child language outcomes than input quantity [21,56]. However, this is a prediction that could be tested in future work by examining whether longer caregiver utterances predict later variation in infant pre-linguistic communication. Second, since infants enjoy engaging in joint attention with others [57,58], it may be that semantically contingent responses provide a richer cue that joint attention has been achieved than responses with more minimal or no lexical content (i.e. by providing further highlighting of the carer’s attentional focus). An alternative explanation along these lines is that the richer content of these responses promotes sustained attention, which has been proposed as a key learning mechanism in communicative contexts [59]. Third, it may also be that, as infants start to understand language, the information obtained from semantically contingent responses is a further motivator, either by being novel to the infant or by matching its expectations regarding the verbal content that its caregiver produces. Given evidence that infants actively seek out new information from informed interaction partners (see e.g. [60]), there may be a positive feedback loop between informationally rich caregiver responses and further infant communication. Previous studies suggest speech plays a role not only in language acquisition but also in broader forms of learning (e.g. pattern recognition, categorization) and social cognition (e.g. understanding others’ goals and communicative intentions) during infants’ first year [61–63], meaning semantically contingent responses may be especially motivating to infants seeking out new information, may bolster existing semantic networks or may provide a strong cue of an opportunity to learn new information [64]. Finally, given the intervention began when infants were around six months, it is worth bearing in mind that differences in infant communication may have emerged because the carers of these infants were engaging in some other scaffolding behaviour(s) prior to 12 months that influenced the development of infants’ communication. For example, rather than being a consequence of an increase in carers’ semantically contingent responding, the effect may have instead been driven by some other change in caregiver responsiveness that we did not measure, such as an increase in vocal feedback to infants’ vocalizations prior to infants turning 12 months. In future research, it would be interesting to sample changes in caregiver interaction over developmental time to establish how early in development interventions might start to influence communicative behaviours, and whether there are any SEC-based differences in infant–caregiver interaction as these interactions first start to emerge.

While caregiver education predicted rates of semantically contingent responses, it did not predict the frequency of carers’ imitative and other vocal responses, nor did it predict infant pre-linguistic communicative acts at 12 months. We are not aware of any previous studies that have examined SEC-based differences in vocal imitation. However, the lack of association of SEC with infant pre-linguistic communication is in line with previous studies of British infants (e.g. [11]). Together, these findings suggest that SEC differences in interaction are specific to those elements involving conventional linguistic content—linguistic input on the part of the caregiver and early words on the part of the infant. When it comes to other aspects of the back and forth of early infant–caregiver exchanges, carers tend to be equally responsive across education categories.

### (a) Strengths and limitations

The study had a number of strengths in its design. It was a pre-registered RCT reported following CONSORT guidelines. The sample, though relying on voluntary participation, was suitably powered, socio-economically diverse (with all participants coming from the lower five deciles of the IMD) and broadly ethnically representative for a UK population (with the caveat that it did not include families who spoke a language other than English at home). By starting the intervention early in infancy (around six months) and collecting data at 12 months, the study was able to address questions regarding the early processes of social interaction and their influence on infants’ earliest pre-linguistic communication. It also provides new insights into SEC-based variation in both caregiver responsiveness and infant pre-linguistic communication at 12 months, with a more specific focus on pre-linguistic communication than previous studies at this age (e.g. [34]). Finally, the use of remote, asynchronously collected video recordings of infant–caregiver interactions is a method that has not been widely employed (see [47]), yet proved capable of providing data with sufficient variance to detect meaningful differences in caregiver responses and infant pre-linguistic communication.

A limitation of the study is that, given the relatively short duration of the recordings, we pooled the pre-linguistic communicative acts identified by Donnellan *et al*. [4] to yield a single measure of pre-linguistic communicative acts rather than examined each behaviour type at the individual level. We therefore cannot draw conclusions about the effects of the intervention on different behaviour types (e.g. show gestures as compared with pointing gestures or gaze-coordinated vocalizations). A further limitation was that the active intervention control was potentially too conservative (in that it involved activities that may have stimulated rich linguistic exchanges, e.g. activities encouraging fine motor skills that may have facilitated talk relating to a variety of objects). Additionally, we do not know precisely how much carers watched or engaged with videos in each condition. It is possible that intervention video content was more engaging to carers and thus more ‘active’ than the active control. Regarding our sample, we had more carers with a degree than without (a 70/30 split), meaning we would need to replicate the effects we report with a larger, more balanced sample to be fully confident that these estimates generalize. Finally, videos were provided on an entirely optional basis. There may be some underlying variable or variables that differentiate the participants who opted to send a video from those in the RCT who did not. For example, these participants may have been more motivated to engage with the study, more confident about appearing on camera or more interested in the intervention (be it the communication intervention or the active control intervention). Likewise, they may have had the time necessary to make the video, or been technically confident in making and sharing a video. The sample was also somewhat weighted towards carers with a degree (although additional analyses controlled for this). Nonetheless, at a very minimum, this tells us (i) whether participants who were willing and able to send in a video had taken on board the intervention messages and acted on them, and (ii) whether this influenced infant pre-linguistic communication.

## 5. Conclusion

Pragmatic language skills and ToM develop from earlier socio-cognitive and communicative skills that are proposed to develop by learning from communicative interaction with carers. This study provides evidence that an early intervention to promote caregiver responsive interaction with infants indeed promoted infants’ early communicative acts. These acts involve the ability to direct others’ attention and as such reflect the communicative roots of later pragmatic language use and the socio-cognitive roots of ToM. It is possible that the semantic content provided by carers is especially valuable at this stage, and further studies should test how this impacts both communication and perspective taking as the two skills grow together. It is our view that any attempt to make sense of the complex relation between pragmatics and ToM must consider the developmental progression from their intertwined origins to their later differentiation, and that understanding their relation requires understanding the learning mechanisms that facilitate this process of separation.

## Data Availability

The data and code supporting this article are accessible via a public Open Science Foundation repository [65]. Supplementary material is available online [66].
